# Percutaneous Irreversible Electroporation: Long-term survival analysis of 71 patients with inoperable malignant hepatic tumors

**DOI:** 10.1038/srep43687

**Published:** 2017-03-07

**Authors:** C. Niessen, S. Thumann, L. Beyer, B. Pregler, J. Kramer, S. Lang, A. Teufel, E. M. Jung, C. Stroszczynski, P. Wiggermann

**Affiliations:** 1 Department of Radiology, University Hospital Regensburg, Regensburg, Germany; 2 Department of Anesthesiology, University Hospital Regensburg, Regensburg, Germany; 3 Department of Surgery, University Hospital Regensburg, Regensburg, Germany; 4 Department of Internal Medicine I, University Hospital Regensburg, Regensburg, Germany

## Abstract

Aim of this retrospective analysis was to evaluate the survival times after percutaneous irreversible electroporation (IRE) in inoperable liver tumors not amenable to thermal ablation. 71 patients (14 females, 57 males, median age 63.5 ± 10.8 years) with 103 liver tumors were treated in 83 interventions using IRE (NanoKnife^®^ system). The median tumor short-axis diameter was 1.9 cm (minimum 0.4 cm, maximum 4.5 cm). 35 patients had primary liver tumors and 36 patients had liver metastases. The Kaplan-Meier method was employed to calculate the survival rates, and the different groups were compared using multivariate log-rank and Wilcoxon tests. The overall median survival time was 26.3 months; the median survival of patients with primary land secondary liver cancer did not significantly differ (26.8 vs. 19.9 months; p = 0.41). Patients with a tumor diameter >3 cm (p < 0.001) or more than 2 lesions (p < 0.005) died significantly earlier than patients with smaller or fewer tumors. Patients with hepatocellular carcinoma and Child-Pugh class B or C cirrhosis died significantly earlier than patients with Child-Pugh class A (p < 0.05). Patients with very early stage HCC survived significantly longer than patients with early stage HCC with a median survival of 22.3 vs. 13.7 months (p < 0.05).

Although hepatocellular carcinoma (HCC) is comparatively rare in Western countries, it is the most frequently occurring solid tumor worldwide, with an annual incidence in excess of one million new patients^1^,^2^,^3^,^4^,^5^,^6^. Apart from primary surgical resection and liver transplantation, there are few curative treatment options for HCC. The survival rates are low for surgical intervention (36–70%) and transplantation (60–70%)^7^,^8^,^9^,^10^. At the time of initial diagnosis, only 10–20% of patients are candidates for surgery and consequently potentially curable. Contraindications for resection generally include multifocal disease, the inability to achieve complete tumor removal and an impaired functional reserve of the liver due to underlying cirrhosis^11^,^12^,^13^. HCC is one of the deadliest human malignancies because there are few curative treatment options for primary, non-resectable hepatic lesions. Of all patients with HCC, 94% will die from the disease^14^.

In Europe and the USA, colorectal liver metastases (CRLMs) are the most frequent cause of malignant liver tumors^15^. Frequently, and particularly in the case of patients with colorectal carcinoma, the liver is the sole site of metastasis^16^. As in HCC, approximately 70–80% of patients with liver metastases are inoperable at the time of diagnosis^17^,^18^,^19^.

Because only a small proportion of patients with liver tumors suffer from an operable tumor disease, new treatment approaches are being investigated to control or even remove liver tumors. In the past two decades, image-guided ablation techniques for focal tumor treatment have attracted substantial attention. Most of these techniques rely on the application of thermal energy for tumor ablation. Irreversible electroporation (IRE) is a non-thermal ablation technique that is currently under early clinical investigation^20^. The aim of this retrospective assessment was to analyze the survival of 71 patients with primary or secondary liver tumors after percutaneous IRE.

## Materials and Methods

For this retrospective study with anonymized patients, approval from the institutional ethical committee was waived. Between October 2011 and July 2015, 71 patients (14 females, 57 males, median age 63.5 ± 10.8 years) with 103 liver tumors were treated via 83 interventions using IRE (NanoKnife^®^ system; Latham, NY, United States) (Table 1). These patients included 35 (49.3%) with primary liver tumors (hepatocellular and cholangiocellular carcinoma) and 36 (50.7%) with liver metastases. The median tumor diameter was 1.9 cm (range 0.4–4.5 cm).The median time period between resection of the primary tumor and the occurrence of liver metastasis was 22.3 ± 14.5 months. Table 2 shows the baseline tumor characteristics.

Each patient was individually discussed within an interdisciplinary tumor board to ensure that all treating physicians agreed with the suggested therapeutic plan. All patients signed a written consent form in accordance with the institutional guidelines. All patients with a primary or secondary liver tumor had no clinical or radiological indications of extrahepatic tumor spread. Patients with cirrhosis of the liver and a related volume of ascites received ascites drainage prior to the start of intervention. Table 3 illustrates the study inclusion/exclusion criteria.

**Figure 1:**
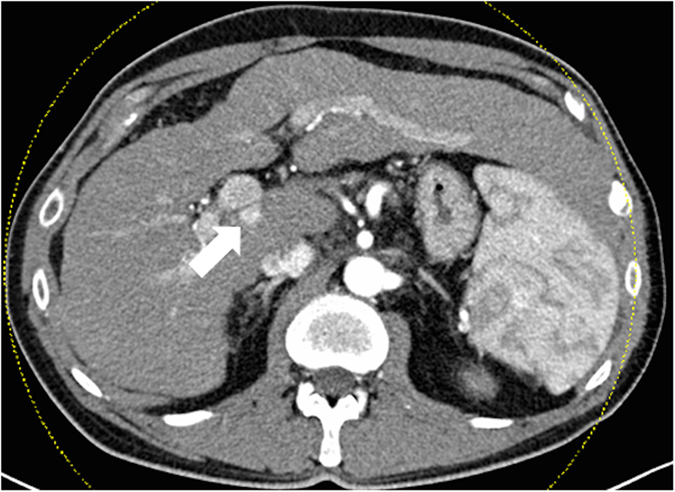
52-year-old patient with hepatocellular carcinoma. Pre-interventional computed tomography for intervention planning: arterial hypervascularized mass on the transition to liver segment I posterior to the main stem of the portal vein.

**Figure 2:**
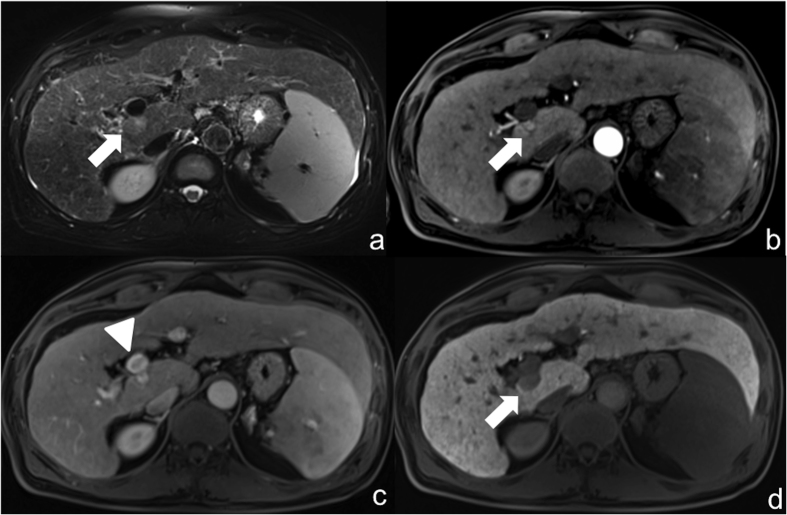
Same patient as in Fig. 1: pre-interventional MRI with liver-specific contrast agent (Gd-EOB-DTPA): (**a**) Hyperintense visualization of the HCC (arrow) in native fat-saturated T2-weighted sequence posterior to the main stem of the portal vein at the transition to liver segment I. (**b**) Dynamic T1-weighted fat-saturated sequence after contrast in arterial phase shows sluggish arterial hypervascularization of the HCC (arrow).(**c**) Dynamic T1-weighted fat-saturated sequence after contrast in portal venous phase shows the directly adjacent main stem of the portal vein (tip of arrow). (**d**) T1-weighted fat-saturated sequence in hepatobiliary phase with wash-out (arrow).

Staging was performed pre-interventionally using computed tomography (CT) of the thorax, abdomen and pelvis (Fig. 1). In addition, MRI of the liver was performed using a liver-specific contrast agent (GD-EOB-DTPA) (Fig. 2).

**Figure 3:**
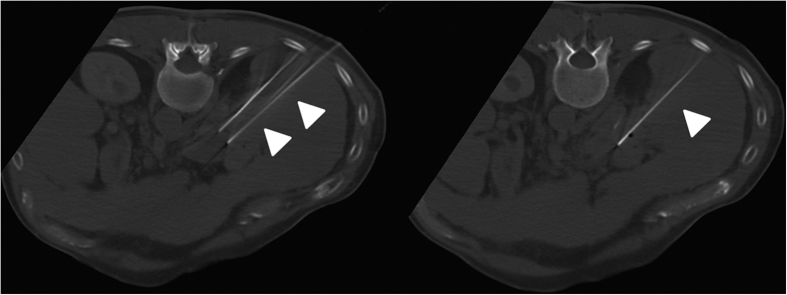
Same patient as in Figs 1 and 2 with HCC. Control CT during irreversible electroporation of HCC mass posterior to the main stem of the portal vein. The intervention required the patient to be placed in prone position in order to insert the 3 electrodes (tips of arrows).

All interventions were performed under general anesthesia and mechanical ventilation with complete muscle relaxation. The electrodes of the NanoKnife^®^ system (Angiodynamics; Latham, NY, US) were percutaneously inserted into all patients using CT fluoroscopy (CareVision, Somatom 16, Siemens, Erlangen, Germany) (Fig. 3).

Depending on the size of the target volume, 2–6 monopolar 18 G ablation electrodes were inserted to completely destroy the tumor and healthy liver tissue within a 1 cm safety margin around the tumor. Accordingly, the length of the tip had to be adapted to the size of the ablation volume (0.5–2.0 cm in 0.5 cm increments). The optimum distance between 2 parallel electrodes enclosing the tumor is between 0.7 and 2.0 cm. Once the correct needle position was verified, a 270 volt test pulse was emitted to ensure adequate conductivity of the tissue prior to initiating the actual ablation algorithm. If conductivity was inadequate, the position of the electrodes must be correspondingly corrected, and the self-test was repeated. The parameters of IRE ablation were 1,650–3,000 V, the pulse length was 90 μs, and 70 pulses were applied per cycle under constant EKG monitoring to avoid life-threatening arrhythmias.

**Figure 4:**
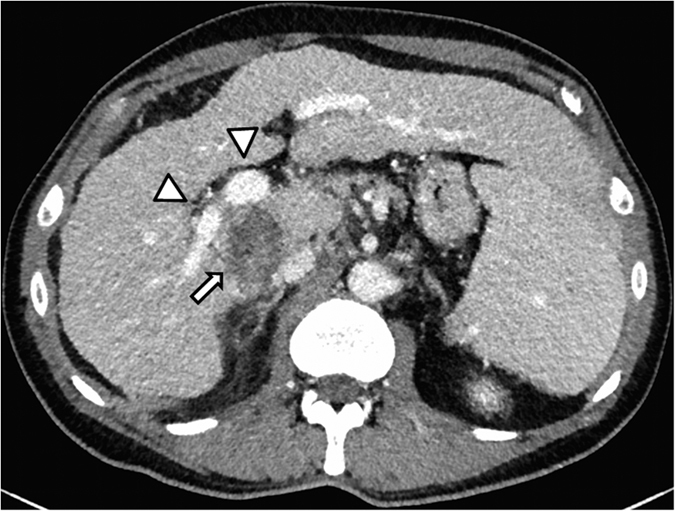
Same patient as in Figs 1, 2 and 3 with HCC. The post-interventional control CT the day after the intervention shows hypodense demarcation of the ablation defect (arrow), and the adjacent portal vein (tips of arrows) is thoroughly contrasted. No post-interventional complications.

To rule out complications, CT and MRI of the liver were performed post-interventionally before the patients were discharged (Fig. 4).

**Figure 5:**
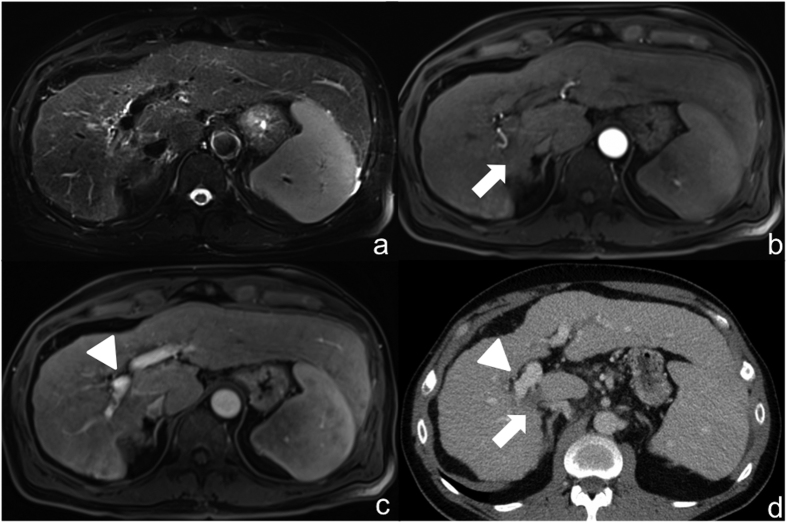
Same patient as in Figs 1, 2, 3 and 4: Follow-up 2 years post-intervention: (**a**) Native, fat-saturated T2 sequence: cicatricial changes after IRE ablation with distinct shrinkage of the ablation defect. (**b**) Dynamic, fat-saturated T2 sequence after contrast: in arterial phase no indication of arterial hypervascularization (arrow), no indication of recurrence. (**c**) Dynamic fat-saturated T1 sequence after contrast: in portal venous phase continued full contrast of portal vein (tip of arrow). (**d**) Follow-up CT in the portal venous phase with full contrast of portal vein (tip of arrow) and distinct shrinkage of the hypodense ablation defect 2 years post-IRE (arrow).

To evaluate the technical success of the intervention, an MRI of the liver was performed 6 weeks post ablation. The actual tumor response was first observed using MRI after 3 months and at 3-month intervals for 2 years after the intervention. Two years post-intervention, MRI scans of the liver were performed two times per year (Fig. 5).

Kaplan-Meier curves were plotted using SPSS (SPSS for Mac, Version 22, Chicago, IL, USA). The Cox proportional hazards model was used for multivariate analysis to evaluate prognostic factors. Factors determining local overall survival were compared using log rank analysis (p < 0.05 was considered significant). The investigated variables were the number of treated lesions, tumor diameter, underlying tumor disease, BCLC and Child-Pugh classification.

## Results

At the end of the study, 36 patients were still alive. Complete ablation, as documented during the 6-week follow-up, was achieved in 95 of 103 lesions (92.2%); 8 lesions required re-treatment due to incomplete ablation (7.8%). The median total survival time was 26.3 months. Local tumor response was not the object of the study. However, after a median follow-up of 35.7 months, 33 of 103 treated lesions (31.7%) demonstrated local recurrence. Therapy-associated side effects were also not the primary aim of this study. However, during 83 interventions, we observed 5 major complications (liver abscess, n = 4; myocardial infarction, n = 1) and 7 minor complications (pneumothorax, n = 2; cardiac arrhythmia, n = 2; hematoma, n = 3). No minor complications required any further treatment.

The median survival of patients with secondary liver tumors was 19.9 months, which was shorter than that of patients with primary liver carcinoma (26.8 months). However, the survival rate did not significantly differ between these two groups (p(LogRank) = 0.41; p(Wilcoxon) = 0.73).

Patients whose tumor was greater than 3 cm (p(Log-Rank and Wilcoxon) < 0.001) exhibited a considerably shorter lifespan. The average survival time of patients with a tumor diameter ≤3 cm was 24.5 months (median survival time was not achieved). The survival time of patients with a tumor diameter >3 cm was 12.9 months (median survival time 9.5 months).

**Figure 6:**
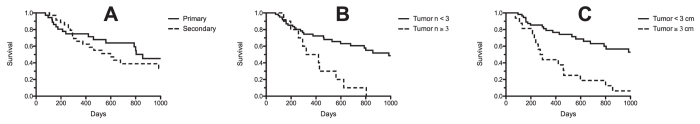
Kaplan-Meier curves: (**A**) The solid line shows the survival time for patients with primary liver tumors (hepatocellular and cholangiocellular carcinoma); the dashed line illustrates the survival time of patients with liver metastases. The survival time of both groups did not exhibit a significant difference. (**B**) The Kaplan-Meier survival curves show significantly better survival for patients with fewer than 3 tumors (solid line) when compared with patients with 3 or more tumors (dotted line). (**C**) Compared to patients with a tumor diameter greater than 3 cm (dashed line), the Kaplan-Meier survival curves show significantly better survival for patients with a short axis diameter less than 3 cm (solid line).

Furthermore, patients with 3 or more lesions demonstrated significantly shorter survival rates (p(Log-Rank) < 0.005; p(Wilcoxon) < 0.005). The median lifespan of patients with no more than 2 lesions was 32.8 months. Those with 3 or more lesions survived for 12.4 months (Fig. 6).

**Figure 7:**
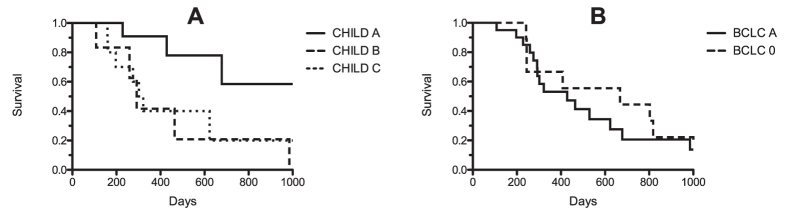
The Kaplan-Meier survival curves for patients with hepatocellular carcinoma: (**A**) significantly better survival of patients with Child-Pugh class A cirrhosis of the liver (solid line) compared to those with Child-Pugh class B (dashed line) and C (dotted line). (**B**) Significantly longer survival of patients with very early stage HCC (dashed line) according to the BCLC classification compared to patients with early stage HCC (solid line).

In a sub-group analysis of patients with HCC (Fig. 7), the survival times of patients with Child-Pugh B or C cirrhosis of the liver were significantly shorter that those in the Child-Pugh A cirrhosis group (p(Log-Rank) < 0.05). Average survival for Child-Pugh A cirrhosis was 19.3 months (median survival time was not reached). In Child-Pugh class B, mean survival was 14.5 months (median: 9.7 months), and in Child-Pugh class C, survival was 12.7 months (median: 10.4 months).

Also, patients with early stage HCC (stage 1) according to the Barcelona Clinic Liver Cancer Classification^21^ (single or max. 3 nodules smaller 3 cm, Child Pugh A, performance status 0) showed significant shorter survival rates in comparison to patients with very early stage (stage 0) HCC (single tumor with a diameter smaller then 2 cm, Child Pugh A, performance status 0): median survival was 22.3 vs. 13.7 months (p < 0.05).

## Discussion

During the past two decades, image-guided percutaneous ablation techniques, such as radio frequency ablation or microwave ablation, have achieved a high level of acceptance, particularly – but not exclusively – with respect to inoperable liver tumors. Various studies have proven that radiofrequency ablation (RFA) is a safe therapeutic option with both low mortality and morbidity^22^,^23^. RFA has shown satisfactory results, with a local post-RFA tumor response rate of over 80% complete tumor ablation in most studies^24^. Likewise, when compared to percutaneous ethanol injection or chemotherapy alone, RFA has demonstrated a significantly higher probability of survival^11^. However, thermal ablation techniques are limited by the so-called heat sink effect. Tumors adjoining larger blood vessels cannot be ablated due to the temperature reduction caused by perfusion. Another limitation of thermal ablation is the risk of thermal damage to the tissue of adjacent structures^25^ or the blood vessels themselves. Typical examples of thermal damage after RFA on the liver are damage to the gallbladder, bile ducts and intestine^26^. Numerous strategies to protect adjoining structures against accidental thermal damage have been described^27^,^28^,^29^. Nevertheless, complete ablation of larger tumors (greater than 3 cm) or ablation of lesions in high-risk locations (such as adjacent to other organs or direct subcapsular position) remains problematic^30^. Several studies of thermal ablation have demonstrated that tumor size and/or an unfavorable (high-risk) site are considered negative prognosis factors for tumor recurrence^31^. The high local recurrence rate in these sites has a negative influence on the long-term outcome and is one of the main reasons thermal ablation is inferior to surgical resection with respect to outcome^32^. For example, Lam *et al*. prospectively treated 298 HCC patients using RFA and demonstrated a significantly shorter survival time for 25 patients whose tumors had been incompletely ablated^33^.

Electroporation is a dynamic phenomenon in which an external electrical field is used to exceed the capacity of the cell membrane, allowing nano-sized pores to be generated in the cell membrane. Depending on the amplitude and duration of the pulse application, electroporation is either reversible or irreversible. IRE results in the loss of cell homeostasis; however, the exact mechanism resulting in cell death remains unexplained. The hypothesis posed by Davalos *et al*. that IRE could be an independent method to ablate soft tissue has been confirmed by subsequent studies of liver cells and in animal models^20^,^34^,^35^. Moreover, the animal model demonstrated that blood vessels and bile ducts within or directly adjacent to the ablation zone remain undamaged^34^. Because thermal ablation techniques are frequently unsuitable for patients with inoperable tumors, chemotherapy frequently remains the sole palliative treatment, thus giving rise to significant interest in a new curative treatment option^36^. For most patients, IRE is currently considered the “last resort” from a therapeutic viewpoint. Likewise, the tumors investigated in this study were inoperable and not treatable using conventional thermal ablation. Nevertheless, an average survival time of 24.3 months was demonstrated for CRLM. This result is promising because chemotherapy would otherwise remain as the only palliative therapeutic alternative for these tumors. After chemotherapy, similar survival times of approximately 18 months have been reported for CLRM in palliative care (fluorouracil with oxaliplatin)^37^,^38^ and 21.7 months for capecitabine, irinotecan and oxaliplatin^39^, but without the burden of therapy associated systemic side effects.

Preclinical studies have demonstrated that IRE creates a well-defined boundary between ablated and non-ablated tissue; thus, the cells are either destroyed or remain intact. Compared with thermal ablation, perivascular tumor ablation with IRE appears to result in less frequent recurrence, indicating that the effectiveness of IRE is not influenced by the heat sink effect^40^. The current state of information does not permit a final statement on IRE. Larger prospective randomized studies will have to confirm these observations. The initial results with smaller hepatic tumors abutting vascular structures and the portal vein are very promising. The success rate is up to 90% but decreases rapidly in relation to tumor size^41^. Our previous study analyzing the risk factors for an early local recurrence demonstrated that similar to conventional (thermal) ablation techniques, a larger tumor diameter represents an independent risk factor for local recurrence^40^. Based on a study of 44 patients, Cannon *et al*. postulated that the best indication for IRE is in the case of tumors with a diameter ≤3 cm that are not accessible using a thermal ablation technique^42^. The results of our study point in the same direction because patients with a tumor diameter >3 cm die significantly earlier than those with smaller tumors (p < 0.001). However, this difference arises primarily because larger tumors are generally associated with greater biological activity and aggressiveness. Thus, larger tumors (diameter greater than 3 cm) may remain the domain of transarterial rather than percutaneous therapy.

In a prospective study, Thomson *et al*. investigated 63 tumors that had been treated using IRE. They found that HCC had distinctly better therapeutic results compared with liver metastases^43^. Likewise, an earlier study by our working group investigated early recurrence after percutaneous therapy using IRE and found that HCC tumors exhibited fewer earlier recurrences compared with other diagnoses^40^. In our current study, patients with HCC demonstrated a longer survival time (26.8 months) compared with those with liver metastases, yet this difference was not comparatively significant. One possible explanation for this phenomenon is that there is different tumor biology between primary and secondary liver cancer leading to different IRE effectiveness.

Overall, it is difficult to draw broad conclusions regarding the impact that percutaneous therapeutic procedures, specifically IRE in our case, have on the total survival time or which additional factors affect treatment using IRE. In addition to this general problem, our analysis has several further limitations, the most important of which is the retrospective nature of the study. Moreover, the patients investigated in the study represent a selected population with distinctly heterogeneous tumor characteristics. In addition, the number of included patients is small, and the follow-up was limited to only 3 years.

Nonetheless, we consider these initial results to be highly promising for the treatment of malignant liver tumors compared with other therapeutic concepts, at least with respect to comparable survival times. Prospective randomized controlled studies with a larger number of patients and longer-term follow-up are required to demonstrate whether IRE, compared with other therapeutic regimes, is superior with respect to survival, local therapeutic outcome and side effects.

## Additional Information

**How to cite this article**: Niessen, C. *et al*. Percutaneous Irreversible Electroporation: Long-term survival analysis of 71 patients with inoperable malignant hepatic tumors. *Sci. Rep.* **7**, 43687; doi: 10.1038/srep43687 (2017).

**Publisher's note:** Springer Nature remains neutral with regard to jurisdictional claims in published maps and institutional affiliations.

## Figures and Tables

**Table 1:** Demographic data.

Variable	Value	
Number of patients	71	
Median age (a)	63.5 ± 10.8	32–84
Gender		
Male	57	80.3%
Female	14	19.7%
Diagnosis		
Hepatocellular carcinoma	31	43.7%
Cholangiocellular carcinoma	4	5.6%
Colorectal carcinoma	27	38.0%
Other metastases	9	12.7%
Tumors treated	103	
Tumors treated per patient	1.5	1–4
Barcelona Clinic Liver Cancer stage for HCC patients		
Stadium 0 (Very early stage)	13	41.9%
Stadium 1 (Early stage)	18	58.1%
T-Stage of colorectal cancer patients at time of diagnosis		
T1	1	3.7%
T2	6	22.2%
T3	14	51.9%
T4a	6	22.2%

**Table 2:** Tumor characteristics.

Variable	Value	
Tumors treated	103	
Tumor entity		
Hepatocellular carcinoma	43	41.7%
Cholangiocellular carcinoma	4	3.9%
Colorectal carcinoma	42	40.8%
Other metastases	14	13.6%
Tumor size		
Median short axis	1.9 ± 0.9 cm	0.4–4.5 cm
Median long axis	2.3 ± 1.0 cm	0.6–5.1 cm
Location		
Segment I	1	1.0%
Segment II, III, IV	50	48.5%
Segment V, VI	25	24.3%
Segment VII, VIII	27	26.2%

**Table 3:** Inclusion/exclusion criteria.

Inclusion criteria	Exclusion criteria
1. Diagnosis of an inoperable primary or secondary liver carcinoma based on biopsy or non-invasive criteria.	1. Any contraindication for general anesthesia
2. Ineligible for conventional thermal ablation due to subcapsular or central tumor location or location adjacent to a major hepatic artery or vein, a bile duct or a major portal vein branch (distance <0.5 cm)	2. Cardiac pacemaker or ICD
3. Age >18 years	3. Vascular invasion, multifocal hepatic disease or extrahepatic tumor manifestations
4. Male or female	4. Prior or present cardiac arrhythmia, myocardial infarction, or significant heart failure
5. Signed consent form	5. Severe coagulation abnormalities (platelet count <50,000/mm^3^; PTT > 50 seconds; INR > 1.5)
